# Status of allele frequency and diversity of *Plasmodium falciparum msp1, msp2* and *glurp* before implementation of an artemisinin-based combined therapy in Northwestern Colombia.

**Published:** 2013-12-31

**Authors:** Amanda Maestre, Eliana Arango, Jaime Carmona-Fonseca

**Affiliations:** Grupo Salud y Comunidad, Facultad de Medicina, Universidad de Antioquia, Medellin Colombia aemaestre@gmail.com

**Keywords:** *Plasmodium falciparum*, malaria, *msp1*, *msp2*, *glurp*, Colombia

## Abstract

**Introduction:**

The status of* msp1, msp2* and *glurp* allele frequency and the diversity of *Plasmodium falciparum *in Northwestern Colombia before the implementation of an artemisinin-combined therapy have been explored only by a few authors and in a relatively small number of samples from this highly endemic region.

**Objective:**

To evaluate the frequency of *msp1, msp2*, and *glurp* alleles and the diversity of* P. falciparum* in two Colombian regions before the use of an artemisinin-combined therapy.

**Methods:**

This study was part of a major anti-malarial efficacy trial designed as a random, clinically-controlled study for which 224 subjects were recruited. Region 2 of *msp1* and *msp2* (central region) were amplified by a nested PCR; *glurp* (region R2) was amplified by a semi-nested PCR.

**Results:**

For msp1, five genotypes were observed, representing the K1, MAD20, and RO33 allelic families. All samples corresponded to a MAD20 150 bp allele. For *msp2* (IC family), two alleles were detected and for glurp, eight were observed. A total 33 haplotypes were detected.

**Conclusions:**

Analysis of *glurp*can be used to successfully genotype parasite populations in the new studies in Colombia aimed at exploring *Plasmodium* spp population dynamics. In addition, analysis of *msp1* and *msp2 *can also be of value for comparisons with past studies, but not when the objective is to study parasites obtained from the same patient in a reduced period of time; for instance, during treatment efficacy studies.

## Abstract (es)

**Introducción:**

La frecuencia alélica y la diversidad de *msp1, msp2* y *glurp* de *Plasmodium falciparum* en el Noroccidente de Colombia antes de la introducción de la terapia combinada con artemisinina, han sido exploradas por pocos autores y en números relativamente bajos de muestras clínicas de esta región de alta endemicidad.

**Objetivo:**

Evaluar la frecuencia alélica y la diversidad de *msp1, msp2* y *glurp* de *P. falciparum* en dos regiones colombianas antes del uso de la terapia combinada con artemisinina.

**Métodos:**

El estudio fue parte de un ensayo mayor sobre la eficacia de la terapia antimalárica con un diseño clínico aleatorio controlado, en el cual 224 sujetos fueron reclutados. La región 2 de *msp1 *y *msp2* (región central) se amplificaron por medio de una PCR anidada; *glurp* (región RII) fue amplificada con una PCR semi-anidada.

**Resultados:**

En msp1, se observaron 5 genotipos que representaron las familias alélicas K1, MAD20, y RO33. Todas las muestras correspondieron al alelo 150 bp de MAD20. En *msp2 *(familia IC), se detectaron 2 alelos y en *glurp,* se observaron 8. Un total de 33 haplotipos fueron detectados.

**Conclusiones:**

El análisis de *glurp* puede ser utilizado para genotipificar exitosamente poblaciones parasitarias en nuevos estudios en Colombia con el objeto de explorar la dinámica de las poblaciones de *Plasmodium* spp. Adicionalmente, el análisis de *msp1 *y *msp2* puede también ser de valor para comparar con estudios pasados, pero no cuando el objetivo es estudiar parasitos obtenidos de un mismo paciente en un periodo reducido de tiempo, por ejemplo, durante estudios de eficacia terapéutica.

## Introduction

*Plasmodium falciparum* parasites evidence genetic diversity in relation to their geographical origin. Several *P.falciparum* genes show extensive genetic polymorphisms and the diversity spectrum has been more fully characterized in parasites from the African continent. *Plasmodium falciparum *represents the second most frequent species after *P. vivax* in the Americas. The highest rates of infection are reported in regions inhabited primarily by persons of African descent. In Colombia, these settlements are along the Pacific coast and in Northwestern Colombia (Uraba, Bajo Cauca and Cordoba regions). 

The genetic characteristics of particular polymorphic genes, such as *msp1, msp2* and *glurp* ^1^ ^-^ ^3^, have been applied to explore parasite population dynamics in malaria endemic areas. In areas of intense transmission, *msp1, msp1* and *glurp* have proved very polymorphic and highly effective in discriminating recrudescent from re-infecting alleles^4^. In the endemic regions of America, variability using these markers has reported uneven results^5^. However, in Colombia, the genes appear disseminated across the country with only minor differences in polymorphism frequencies^6^ ^-^ ^8^. Major climate changes have affected our country during the past 10 years. This has been seen mainly by extremes in the dry and rainy seasons that have resulted from the El Niño/Southern Oscillation (ENSO)^9^. This can have severe consequences on the prevalence and dissemination of particular genotypes. For example, resistant clones might easily spread through regions that share the same eco-epidemiological area after there have been major changes in mosquito populations and intensity rates.

In addition, the implementation in 2006 of a nationwide policy of artemisinin-combined therapy against falciparummalaria resulted in a new challenge for health authorities: to provide rapid diagnoses and effective treatments in a country where political instability is widespread in the malaria endemic regions.

Treatment with artemisinin-based schemes further affects the genotype of *P. falciparum* populations by introducing new drug pressures on parasites already resistant to other anti-malarials^10^. In this context, exploration of the genetic characteristics of this species has been a major focus of our research.

In order to establish the baseline genetic composition of *P. falciparum,* clinical isolates were identified before the introduction of artemisinin-combined treatments from the regions with the highest frequency of malarial infection in the country. In order to provide adequate genetic information for future studies that will explore changes in population structures, we are reporting the genotypes of all archived samples collected from 2004 to 2008 in Northwest Colombia. This was done as part of a nationwide study aimed at monitoring the presence of anti-malarial resistance.

## Materials and Methods

### Study sites and sample design

This study was part of a major anti-malarial efficacy trial that took place in the municipalities of Necocli (8º,25´ 11´´ N, 76º 45´ 58´´ W), Turbo (8° 5´4´´ N, 76° 44´ 123´´ W), El Bagre (7° 35´ 2´´ N, 74° 48´ 2´´ W) and Zaragoza (7° 29´ 2´´ N, 74° 51´ 35´´ W). These are located in two regions of Northwestern Colombia where malaria is highly endemic: Urabá and Bajo Cauca. Both regions have: a) high migration rates and perennial malarial transmission that was unstable with mean annual parasite index (API: numbers of cases per 1,000 inhabitants) - during 2004-2008 of 61.47 in Necocli, 33.09 in Turbo, 128.48 in El Bagre and 127.04 in Zaragoza^11^ ^-^ ^13^; b) these are mainly inhabited by African descendants and persons with indigenous ancestry, as well as by those with mixed indigenous and Spanish ancestry; c) the economy of Bajo Cauca is based on gold mining, whereas Urabá is a banana-producing region^14^ ^,^ ^15^.

The macro-project was designed as a random, clinically-controlled study in which anti-malarial efficacy was assessed on the basis of the mean incidence of falciparum malaria for each 10,000 cases per year (50% in each region) with an estimated 20% maximum treatment failure rate, 95% confidence interval, 5% sampling error. The current report presents the results for all subjects recruited in the original study from which genetic material was available.

The study included patients attending the local malaria clinics with an acutely symptomatic, non-severe form of *P. falciparum* malaria. Unique infection by this species was confirmed by microscopy and by nested PCR. The inclusion criteria for the original study were: ≥1 year of age, unique *P. falciparum* parasitaemia of ≥1,000 asexual forms/µL, and the willingness to participate. Patients were excluded if consent was withdrawn.

The study protocol was reviewed and approved by the Ethics Committee of the Faculty of Medicine, Universidad de Antioquia (Medellín, Colombia). Each participant gave their fully informed consent. 

### Diagnosis of *P. falciparum* infection

Whole blood was taken from a peripheral vein on day 0 before the administration of treatment. Giemsa-Field stained thick/thin blood films were examined at 100x magnification to identify the presence of parasites, *Plasmodium* species, parasite density, and schizontaemia.

Parasite density was measured by counting the number of asexual parasites per 200 leukocytes based on a mean count of 8,000 leukocytes per microliter of blood (theoretical value)^16^. A slide was considered negative after the examination of at least 300 microscopic fields^16^. In addition, a nested PCR was performed to confirm unique infection by *P. falciparum*. Amplification from whole blood, collected and extracted (as detailed in the Molecular genotyping section), was carried out using a nested PCR assay to detect the 18s ribosomal RNA (rRNA) gene of *P. falciparum* and *P. vivax*, according to previously published procedures^17^. Amplification products were resolved in a 2% agarose gel using ethidium bromide and viewing under UV light. 

### Molecular genotyping

Only pre-treatment samples (day 0) from sequentially recruited subjects were selected for the genotyping of *msp1, msp2, *and *glurp.*

A volume of 100 mL of whole blood was placed on Whatman 3MM filter paper and stored at -20^○ ^C until DNA extraction with Chelex^(r)^. Briefly, a piece of filter paper was soaked overnight in a solution of 10% saponin in PBS and was subsequently washed in PBS. Afterwards, genomic DNA from each sample was obtained by boiling, followed by ethanol precipitation that was conducted according to established procedures^18^.

The region 2 of *msp1* and *msp2* (central region) were amplified by a nested PCR; *glurp* (region R2) was amplified by a semi-nested PCR^19^. Products obtained after the first PCR, were amplified using specific primers for region 2 of *msp1* corresponding to MAD20, K1, and RO33 allellic families, and FC27 and IC-1 for the central region of *msp2*. The sequence of the primers and the protocol of PCR are described in detail by Snounou *et al* ^19^. Briefly, a PCR was carried out in a total volume of 20 µL, containing 10 mM Tris-HCl (pH 9.0 at 25^○ ^C), 50 mMKCl 134 and 0.1% Triton X-100, 125 mMdNTPs, 0.4 units Taq DNA polymerase (Fermentas), 1.6 mM MgCl_2_, and 125 nM of each primer. Initial denaturation was for 5 min at 95^○ ^C, 1 min at 94^○ ^C, 2 min at 58^○ ^C annealing (all first PCRs and secondary reactions for *glurp*) or at 61^○ ^C (for all the secondary reactions of *msp1* and *msp2*). This was followed by an extension for 2 min at 72^○ ^C. This first reaction underwent 25 amplification cycles and the secondary 30 cycles. 

Positive (strains HB3, K1, and RO33) and negative controls (healthy individuals) were included. Products were electrophoresed on agarose (MetaPhor) gel (2.5% for *msp1* and 2% for *msp2* and *glurp*), stained with ethidium bromide, and read under ultraviolet light. Size polymorphism for each gene was assessed using the Quantity One (Bio-Rad) program. 

### Statistical analysis

Frequencies were calculated for each municipality and for the total number of samples. The frequency of multiple infections was calculated based on the presence of two or more bands of the same marker in one sample. This was estimated for each marker and for each municipality. 

## Results

In total, genetic material was available from 224 subjects from the two regions: 136 in Uraba (Turbo n= 95, Necocli n= 41) and 88 in Bajo Cauca, (El Bagre n= 75, Zaragoza n= 13). From this sample, all (100%) were successfully amplified for *msp1*, 205 (91%) for *msp2* and 172 (77%) for *glurp*. 

### Genetic diversity and allelic frequency 

For *msp1*, five different genotypes were observed: representing the K1 (2 genotypes), MAD20 (1 genotype) and RO33 (2 genotypes) allelic families. The *msp1* fragment sizes ranged from 150-270 bp. However, all samples corresponded to the monomorphic MAD20 150 bp genotype (Table 1) while the K1 (150-200 bp) and RO33 (250-270 bp) allelic families were detected below the level of 5%. 

**Table 1:**
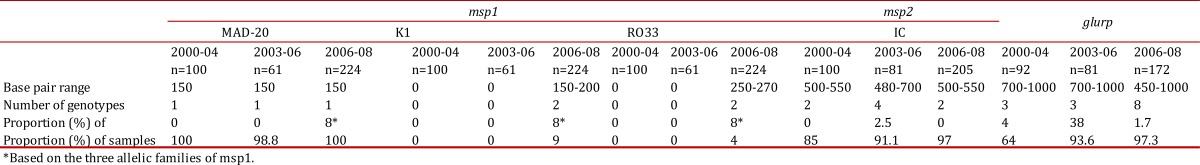
Comparison of genotypes according to the msp1, mps2, and glurp genes in the four localities examined. Results are compared according to the year of sample collection as reported by different authors in the periods of 2000-2004, 2004-2006 20-23, and 2006-2008 (the current study). Alleles corresponding to the FC27 family of msp2 were absent in all studies

A total of 2 different *msp2* genotypes (size 500 and 550 pb) were recorded in 91% of the samples and they were all from the IC allelic family. In this marker, an 85% frequency rate was detected for the fragment of 500 bp.

**Figure 1:**
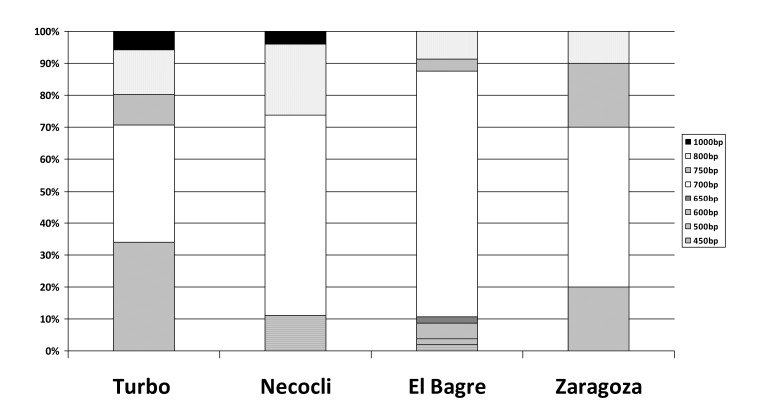
Detail of the polymorphism size of the glurp gene in each locality. Frequencies for the amplified fragments, according to the size of the are presented for each locality.

The frequencies of the individual *glurp* genotypes were high. In total, eight different genotypes were detected. The allelic variants ranged between 450 and 1,000 bp, and most samples exhibited the 700 bp sized fragment (58.1%), followed by the 600 (16.2%) and 800 (14.5%) bp fragments (Fig. 1). 

Mean multiplicity of infection (MOI), calculated using data from each of the three marker genes, was 0.023 for *msp1*, 0.027 for *msp2*, and 0.047 for *glurp*.

**Figure 2:**
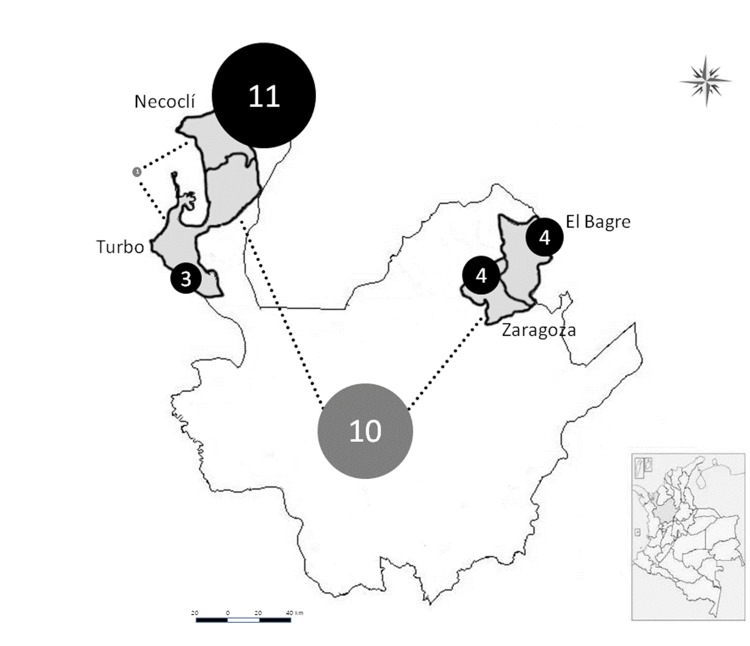
Frequencies of exclusive and shared haplotypes according to the locality examined. Analysis based on msp1, msp2, and glurp. Each number represents particular genotypes; grey circles: shared haplotypes; black circles: exclusive haplotypes.

A total 33 haplotypes were detected with a predominance of exclusive haplotypes in each of the localities studied. The highest number of exclusive haplotypes was observed in Necocli (n= 11), and the lowest number was detected in Turbo (n= 3). Shared haplotypes in the two main regions (Uraba and Bajo Cauca) were frequently found (Fig. 2).

## Discussion

Polymorphic regions of the *P. falciparum *genes* msp1, msp2* and *glurp* loci have been selected as the recommended markers for parasite genotyping in anti-malarial drug trials and efficacy studies^4^. However, the parasites' genetic profile has not been systematically documented in Colombia. In this study, a large number of archived *P. falciparum* positive pre-treatment parasites were genotyped in order to compare the diversity and allelic frequencies for these in two regions of Colombia exhibiting the highest levels of endemicity. 

Homogeneity of *P. falciparum* populations was confirmed in these regions of Colombia, according to the genes studied. This is in striking contrast to observations made in the parasites from Africa where *msp1, msp2* and *glurp* markers were highly polymorphic and had low allele frequencies^4^. These observations have been consistent overtime, as confirmed by diverse studies performed in the region from the works studies of 2000-2001^20^. In Table 1, a comparison is made of the different studies reported with the samples taken in Colombia. This illustrates an apparent reduced polymorphism based on an analysis of these genes, regardless of the diverse *P. falciparum* endemicity^21^ ^,^ ^22^. Homogenous *P. falciparum* populations, according to *msp1* and *msp2* after fixation of MAD-20 alleles, have also been reported by other authors (Table 1)^22^ ^,^ ^23^. However, a recent emergence of K1 and RO33 alleles is evidenced from the current study. Several factors might account for this phenomenon, such as the introduction of new alleles after migration events and/or the parasite´s selection after the introduction of new treatment schemes. The latter appears to be the case in Colombia since from 2006. 

Genetic diversity is suggested by the differences in the amplified fragment size of the alleles detected for* msp1* and *msp2 *from the different studies carried out in Colombia. This finding can be interpreted as allele variation or allele evolution. However, the application of different methodologies across these studies makes comparisons difficult, as suggested by Färnert *et al* ^24^. It is recommended that a unified system be used to explore variability in Colombian samples. In any case, continuous monitoring of the presentationof *msp1* alleles is worthy of pursuit in order to confirm and early define changes in the population's structure.

For *glurp*, a trend of diagnosing larger number of alleles has been detected over time and this is in direct relationship with endemicity and the number of samples evaluated^21^. This was also confirmed in the present study. Nevertheless, the number of alleles detected by this marker remains significantly low compared to African parasites^4^. Furthermore, the frequency of multiplicity of infection using the selected marker genes was low when compared with the African parasites ^4^. However, in the present study, a high frequency of polyclonal infection using *glurp* was detected during 2004-2006. Thereafter, it returned to lower levels. This can be explained by particular climatic phenomena affecting mosquito populations which may select for particular parasitic genotypes and subsequently change the transmission pattern. Alternatively, this may be the result of a natural process that was halted by the introduction of new anti-malarial treatments. 

Based on these results we conclude that an analysis of *glurp* might be used successfully to genotype parasitic populations during new studies in Colombia aimed at exploring parasite population dynamics and the *in vivo* efficacy of anti-malarials. In addition, analysis of *msp1* and *msp2* can also be of value to make better comparisons with past studies, but not when the objective is to study parasites obtained from the same patient in a reduced period of time; for example, during treatment efficacy studies. Sequencing analysis of these genes in Colombian samples might prove useful to understand population dynamics in the different regions of the country and to further confirm the homogeneity of the populations.
